# Polyphenol-rich extract from grape and blueberry attenuates cognitive decline and improves neuronal function in aged mice

**DOI:** 10.1017/jns.2018.10

**Published:** 2018-05-21

**Authors:** Julien Bensalem, Stéphanie Dudonné, David Gaudout, Laure Servant, Frédéric Calon, Yves Desjardins, Sophie Layé, Pauline Lafenetre, Véronique Pallet

**Affiliations:** 1 Université de Bordeaux, Nutrition et Neurobiologie Intégrée, UMR 1286, Bordeaux, France; 2 Institut national de la recherche agronomique (INRA), Nutrition et Neurobiologie Intégrée, UMR 1286, Bordeaux, France; 3 Activ'Inside, ZA du Grand Cazau, 33750 Beychac et Caillau, Région de Bordeaux, France; 4 Institute of Nutrition and Functional Foods (INAF), Laval University, Quebec, Canada; 5 OptiNutriBrain International Associated Laboratory (NutriNeuro France–INAF Canada); 6 Université Laval, Faculté de Pharmacie, Québec, QC, Canada; 7 Bordeaux INP, Nutrition et Neurobiologie Intégrée, UMR1286, Bordeaux, France

**Keywords:** Berries, Polyphenols, Ageing, Cognitive decline, Hippocampus, Neurogenesis, *Bdnf*, brain-derived neurotrophic factor, DCX, doublecortin, DG, dentate gyrus, IR, immunoreactive, MWM, Morris water maze, *Ngf*, nerve growth factor, NOR, novel object recognition, PEGB, polyphenol-rich extract from grape and blueberry

## Abstract

Ageing is characterised by memory deficits, associated with brain plasticity impairment. Polyphenols from berries, such as flavan-3-ols, anthocyanins, and resveratrol, have been suggested to modulate synaptic plasticity and cognitive processes. In the present study we assessed the preventive effect of a polyphenol-rich extract from grape and blueberry (PEGB), with high concentrations of flavonoids, on age-related cognitive decline in mice. Adult and aged (6 weeks and 16 months) mice were fed a PEGB-enriched diet for 14 weeks. Learning and memory were assessed using the novel object recognition and Morris water maze tasks. Brain polyphenol content was evaluated with ultra-high-performance LC-MS/MS. Hippocampal neurotrophin expression was measured using quantitative real-time PCR. Finally, the effect of PEGB on adult hippocampal neurogenesis was assessed by immunochemistry, counting the number of cells expressing doublecortin and the proportion of cells with dendritic prolongations. The combination of grape and blueberry polyphenols prevented age-induced learning and memory deficits. Moreover, it increased hippocampal nerve growth factor (*Ngf*) mRNA expression. Aged supplemented mice displayed a greater proportion of newly generated neurons with prolongations than control age-matched mice. Some of the polyphenols included in the extract were detected in the brain in the native form or as metabolites. Aged supplemented mice also displayed a better survival rate. These data suggest that PEGB may prevent age-induced cognitive decline. Possible mechanisms of action include a modulation of brain plasticity. Post-treatment detection of phenolic compounds in the brain suggests that polyphenols may act directly at the central level, while they can make an impact on mouse survival through a potential systemic effect.

Whether dietary factors or changes in lifestyle help to prevent or delay age-related decline in brain function, starting in midlife, remains an important question^(^^1^^,^^2^^)^. Among foods beneficial to the brain, fruits and vegetables rich in polyphenols have been reported to postpone age-related physiological and functional deficits^(^^3^^,^^4^^)^. Polyphenols form complex families of compounds, exclusively synthesised in the plant kingdom. The main families are flavonoids, phenolic acids, stilbenes, lignans and tannins^(^^5^^)^. Previous studies have shown that daily consumption of polyphenol-rich blueberry or grape juice for 12 weeks improved episodic memory performances in elderly subjects^(^^6^^,^^7^^)^. More recently, we found that the combination of a polyphenol-rich extract from grape and blueberry was able to prevent age-related memory impairment in middle-aged mice (16 months old) after 8 weeks’ supplementation^(^^8^^)^. The synergistic potential of grape and blueberry phenolic compounds was highlighted in a recent bioavailability study, reporting that blueberry phenolic metabolites increased in mouse plasma when co-ingested with polyphenol-rich grape extract. The same study also revealed that a chronic administration of the grape–blueberry combination significantly increased plasma phenolic concentrations, compared with a single acute dose. This finding was not observed with individual extract supplementation^(^^9^^)^.

The neurobiological mechanisms of action of polyphenols remain unclear, but recent evidence suggests that they modulate cell and molecular processes involved in learning and memory, including neuronal signalling pathways involved in synaptic plasticity^(^^10^^)^. Key polyphenols, like the flavan-3-ols, anthocyanins and stilbenes present in grapes and blueberries have also been shown to increase markers of hippocampal neurogenesis in rodent models^(^^11^^,^^12^^)^. The modulation of the expression of genes involved in memory processes, such as calmodulin-dependent kinase II and nerve growth factor (*Ngf*), by a grape–blueberry combination has already been suggested in middle-aged, but not yet in aged mice^(^^8^^)^. It remains to be determined whether a combination of grape and blueberry extracts exhibits all these effects during ageing, as negative interactions could result in less marked activity^(^^13^^)^.

The aim of the present study was to further assess the behavioural benefits of the polyphenol-rich extract from grape and blueberry (PEGB) in aged mice (16 months old) supplemented for 14 weeks (12 weeks before the behavioural tests). Phenolic compounds were analysed in the brain to evaluate PEGB brain bioavailability. The global preventive role of PEGB was also evaluated by monitoring the survival rate of mice that completed the supplementation course. We investigated the mechanisms responsible for the beneficial effect of PEGB, i.e. adult hippocampal neurogenesis and neurotrophic factor expression (brain-derived neurotrophic factor (*Bdnf*) and *Ngf*), on long-term memory, mainly targeting hippocampal correlates, as the integrity of the hippocampus is essential for proper functioning of long-term memory and particularly affected by ageing.

## Materials and methods

### Animals

For the present study, sixty-seven adult (6 weeks old) and sixty-seven aged (16 months old) male C57Bl/6J mice were purchased from Janvier. The mice were given *ad libitum* access to food and water. All experiments were performed in accordance with the European Communities Council Directives (86/609/EEC) and the French National Committee (87/848) recommendations, and approved by the Animal Care and Use Committee of Bordeaux (no. 5012085-A).

### Intervention diet and polyphenol-rich extract

Mice were randomly divided into four experimental groups. One group of adult mice and one group of aged mice were fed with a control diet (INRA), whereas the other two groups of adult and aged mice received a polyphenol-enriched diet (INRA) at a dose of 500 mg of PEGB/kg body weight per d. In a double-blinded, randomised, placebo-controlled clinical study, subjects (from France and Canada) received 600 mg/d of the same PEGB. We observed a positive effect of this dose on episodic memory of aged subjects in a subgroup of the study population that exhibited the highest age-related cognitive decline (data not shown). This amount could reasonably be achieved in the human population. The composition of the control diet was the same as the polyphenol-enriched diet, except for its polyphenol content (Supplementary Table S1). The diet, as well as the PEGB dose and composition were the same as those used in previous studies^(^^8^^,^^9^^,^^14^^)^. The diet started as soon as mice arrived in the laboratory (i.e. at the age of 6 weeks for adult mice and 16 months for aged mice) and continued throughout the entire experiment, i.e. 14 weeks: 12 weeks before behavioural evaluation plus 2 weeks during the tests (6 weeks for bioavailability measurements). The diet was renewed twice per week. The polyphenol-rich extract (provided by the Neurophenols Consortium) is a powder made of grape (*Vitis vinifera* L.; Activ'Inside) and blueberry (*Vaccinium angustifolium* Aiton; NutraCanada) extracts, containing specific low-molecular-weight polyphenols. It was mainly constituted of flavan-3-ols, particularly monomeric catechin and epicatechin, provided by the grape extract and flavonols (as quercetin), anthocyanins and chlorogenic acid provided by the blueberry extract. A brief in-house analysis of the polyphenol content is presented in Table 1. The polyphenol stability in the diet was monitored over the supplementation period.

**Table 1.** Intake of phenolic compounds in mice supplemented with polyphenol-rich extract from grape and blueberry

	Dose (mg/kg BW)
Flavan-3-ols (total)	205·5
DP 1–3	171·0
(+)-Catechin + (−)-epicatechin	128·7
DP > 3	34·5
Anthocyanins	2·2
Stilbenes (resveratrol)	0·2
Flavonols	4·5
Phenolic acids	18·2
Total*	297·5

BW, body weight; DP, degree of polymerisation.

* Total phenolic intake was determined using the Folin–Ciocalteu assay.

### Polyphenol bioavailability in brain

In order to evaluate the presence of polyphenols in the brain, a batch of six control mice (three adult and three aged) and twenty supplemented mice (ten adult and ten aged) were fed with the control or PEGB-enriched diet for 6 weeks. Mice were then euthanised by cervical dislocation and decapitated. Brains were dissected, washed with 0·9 % sodium chloride, frozen with liquid N_2_, and then stored at −80°C until assay.

#### Extraction and characterisation of phenolic metabolites from brain tissues

Phenolic metabolites were extracted from brain tissues and characterised using ultra-high-performance (UHP) LC-MS/MS, as previously described^(^^14^^)^. The brains were not perfused to wash out the blood because this procedure altered the chemical equilibrium between the vascular compartment and the whole brain^(^^15^^)^. Briefly, frozen brains were mixed with 4 % phosphoric acid (material:solvent ratio, 1:4) and ground with glass beads using a Biospec BeadBeater for 15 s, then the homogenate was centrifuged at 15 000 rpm at 4°C for 15 min. Phenolic metabolites were extracted from the supernatant fraction using Waters OASIS HLB 2 mg 30 µm micro-elution plates. Eluted phenolic compounds were directly analysed by UHPLC-MS/MS, specifically targeting aglycones and conjugates of (epi)catechin, resveratrol and quercetin, as well as their main microbial metabolites, i.e. valerolactones and derivatives of cinnamic, benzoic, propionic and acetic acids. MS/MS analyses were carried out in negative mode and data were acquired in the multiple reaction monitoring mode, tracking the specific parent product ion transition for each compound. Cone voltage and collision energy parameters were optimised for each compound. The metabolites were identified by comparing their retention times and molecular ions with those of available phenolic standards (catechin and epicatechin; Sigma-Aldrich) and the quantification was conducted using their calibration curves. Metabolites for which standards were not available (methyl catechin glucuronide, catechin glucuronide) were identified based on fragmentation information described in the literature and quantified using the calibration curve of epicatechin. Limits of detection and quantification of identified compounds were 0·3 and 1 pmol/g, respectively. A null value was attributed when no polyphenol was detected by the method (no quantification available).

### Survival monitoring

At the beginning of the study, 108 mice were followed for survival monitoring. Mouse deaths from natural causes and the date of death were recorded during the experiment. A total of fifty-four adult and twenty-seven aged mice were followed for 14 weeks. Thus, adult mice were monitored from 6 weeks to 5 months of age and aged mice from 16 to 19·5 months of age. At the end of the 14-week dietary supplementation, the remaining mice were euthanised for biological measurements.

### Behavioural evaluation

We examined the effects of 14-week PEGB supplementation on novel object recognition (NOR) and spatial learning and memory in the Morris water maze (MWM). Twenty mice per group were assigned to these tasks.

#### Novel object recognition

The experimental apparatus consisted of a white rectangular open field (45 cm × 25 cm × 40 cm). During the first session, mice were habituated to the open field in the absence of objects for 10 min. Mice were then returned to their home cage for 15 min before the second session. During the second session, mice were placed in the same open field but with two identical objects (two squares or two pillars). The objects were counter-balanced between mice to control object preference. Mice were allowed to freely explore the environment and objects for 10 min. After 24 h, mice were placed back in the open field for the testing phase. At this time, the two objects were replaced by a novel one and a triplicate (familiar object) of the training session. The positions of the novel and familiar objects were counter-balanced between mice to avoid innate preference for a location. Mice were allowed to explore them for 15 min. Preference for the novel object was expressed as the percentage time spent exploring the novel object compared with the percentage time spent exploring the familiar object. Mice were considered to be exploring the objects when they were facing them in very close proximity, sniffing them, and/or touching them. Each phase was video-recorded and exploration time was measured using an event programme.

#### Spatial learning and memory in the Morris water maze

Mice were tested in an MWM, as described in Bensalem *et al.*^(^^8^^)^. Mice were first familiarised with water and swimming during two familiarisation days (three consecutive trials per d; 60 s-cut-off), when they had to find a visible platform in the centre of a small pool (60 cm diameter) surrounded with curtains. Then, to evaluate visuomotor deficits, mice were tested in a cued task, where they were given six trials (90 s cut-off) to find a visible platform identified with a cue in one of the four quadrants of the MWM, surrounded with curtains. During the training sessions, the animals were required to locate the submerged platform, placed in a new quadrant of the pool different from the visible platform test, by using distal extramaze cues. They were trained for six trials per d (90 s cut-off), with an intertrial interval of 5 min, for 5 consecutive days. In order to facilitate spatial learning, mice were introduced from four different starting points, in a randomised daily order. Speed, latency and distance to reach the platform were recorded by the Imetronics videotrack system.

Spatial memory was evaluated 48 h after the last training session during the probe test. The platform was removed from the pool and spatial memory was evaluated for 60 s. The percentage of time spent in the quadrant where the platform had been located during training (target quadrant) and the number of the target annulus crossings were recorded using the Panlab SMART system (Bioseb).

### Tissue preparation

At 90 min after the probe test, mice were euthanised by cervical dislocation and decapitated. Half of the brains were dissected and hippocampal mRNA expression was measured by quantitative real-time PCR. The remainder was dissected for hippocampal neurogenesis analysis by immunohistochemistry. For PCR analysis, hippocampi were rapidly dissected and frozen in liquid N_2_ and then stored at −80°C until assay. In order to analyse hippocampal neurogenesis by immunohistochemistry, the dissected brains were washed and immersed in 4 % paraformaldehyde. After a 3-week post-fixation period, 50 µm coronal sections were cut on a vibratome (Leica).

### Real-time PCR analysis of gene expression in the hippocampus

Hippocampal gene expression was measured, as previously described^(^^8^^,^^16^^)^. Briefly, RNA was extracted using TRIzol reagent (Invitrogen). RNA concentrations were determined using a Nanodrop ND-1000 (Labtech). cDNA were synthesised from 1 µg of RNA with ImPromII reverse transcriptase (Promega), using oligodT and random primers (Promega).

Real-time PCR was performed using the LightCycler 480 system with a ninety-six-well format (Roche Diagnostics). The forward- and reverse-primer sequences and the amplicon size for glyceraldehyde-3-phosphate dehydrogenase (*Gapdh*), *Ngf* and *Bdnf* are summarised in Supplementary Table S2. *Gapdh* was used as the reference gene, since its expression level was unaffected under our experimental conditions.

Quantification data were analysed using LightCycler 480 Relative Quantification software (version 1.5). Therefore, the results are expressed as the target:reference ratio divided by the target:reference ratio of the calibrator. In our case, the calibrator was chosen among the adult mice.

### Immunohistochemistry

Free-floating sections were processed with a standard immunohistochemical procedure^(^^17^^)^. A one-in-twelve section (50 µm) was treated for doublecortin (DCX) immunoreactivity using a goat polyclonal antibody (1:1000; Santa Cruz Biotechnology) and a biotinylated donkey anti-goat secondary antibody (1:200; Amersham). All sections were processed in parallel and immunoreactivities were visualised by the biotin–streptavidin technique (ABC kit; Dako), using 3,3-diaminobenzidine as chromogen. The number of immunoreactive (IR) cells in the left dentate gyrus (DG) was estimated using a modified version of the optical fractionator method, with systematic random sampling of every twelve sections along the rostro-caudal axis of the DG. On each section, IR cells in the granular and subgranular layers of the DG were counted, by an experimenter blinded to the group assignments, with a 40× microscope objective (Mercator software)^(^^17^^)^. Among these, the IR cells with prolongations were quantified. Results are expressed as the total number of DCX-IR cells in the whole DG or the percentage of DCX-IR cells with dendrites.

The gene expression and the number of IR cells were measured on the animals subjected to the behavioural study.

### Statistical analysis

Survival was analysed using χ^2^ analysis on the Kaplan–Meir survival curves. Swimming speed, probe test comparisons, PCR and neurogenesis data were analysed using a two-way ANOVA (effect of age and diet) followed by a *post hoc* Fisher protected least significant difference (PLSD) test. Probe test results of each group were also compared with chance level (25 %) using a one-sample *t* test. NOR and spatial learning were analysed using a three-way ANOVA with repeated measures (effect of age, diet, and, respectively, objects or days) followed by a *post hoc* paired *t* test for the NOR and a Fisher PLSD test for the spatial learning. Data in the figures are expressed as mean values with their standard errors. Results were considered significantly different when *P* < 0·05.

## Results

### Brain bioavailability of polyphenols

In order to determine whether ingested polyphenols reached the brain, its polyphenol composition was analysed by UHPLC-MS/MS. As flavan-3-ols were the most widely represented polyphenol family in PEGB (Table 1), their native forms (catechin and epicatechin) and conjugated metabolites (methylated, glucuronidated and sulfated conjuguates) were analysed. Aglycones catechin and epicatechin, catechin glucuronide and methyl catechin glucuronide were detected in the brain in both adult and aged supplemented mice, with no inter-age differences. It is noteworthy that no polyphenols were found in mice fed with the control diet (Table 2).

**Table 2.** Flavanol metabolites identified in the brains of mice after 6 weeks of supplementation with polyphenol-rich extract from grape and blueberry (PEGB) (Mean values of replicates with their standard errors)

	Brain content (pmol/g)					
	Adult control (*n* 3)	Adult PEGB (*n* 10)		Aged control (*n* 3)	Aged PEGB (*n* 10)	
Metabolite	Mean	Mean	sem	Mean	Mean	sem
(−)-Epicatechin	nd	3·54	1·58	nd	8·84	4·87
(+)-Catechin	nd	0·88	0·47	nd	7·07	3·32
Methyl catechin glucuronide	nd	5·60	2·71	nd	3·15	1·12
Catechin glucuronide	nd	0·81	0·59	nd	1·39	0·61

nd, Not detected.

### Effects of age and polyphenol-rich extract from grape and blueberry-enriched diet on survival

**Fig. 1.**
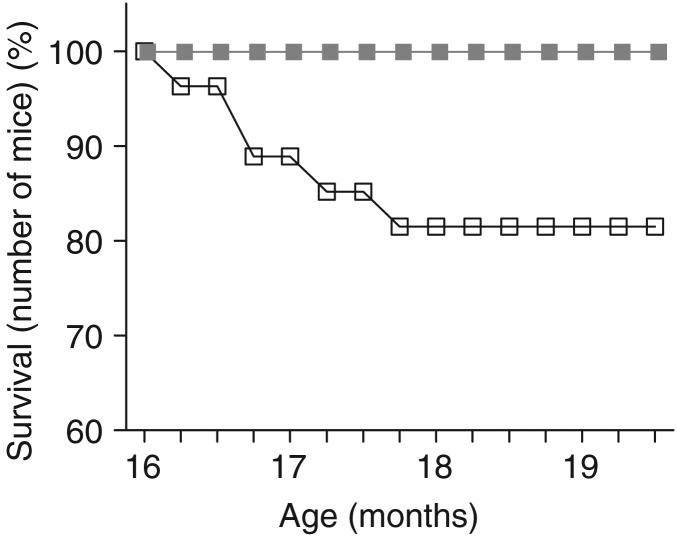
Survival curve of aged mice from the beginning until the end of the 14-week supplementation diet. An absence of mortality in aged mice supplemented with polyphenol-rich extract from grape and blueberry (PEGB; 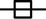) during this observation period contrasted with almost 20 % mortality of aged mice on the control diet (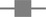) (χ^2^ analysis on Kaplan–Meir survival curves: *P* < 0·05; *n* 27 per group).

Survival rates were monitored in twenty-seven mice per group from the beginning until the end of supplementation. No adult mice died during the experimental period, irrespective of diet (control or PEGB-enriched). Thus, we focused our analysis on aged mice. A χ^2^ analysis on the Kaplan–Meir survival curves revealed a difference in mortality between control and supplemented aged mice (df = 1, χ^2^ = 5·41, *P* = 0·02). Our results revealed that 18·52 % of aged mice (five of twenty-seven mice) fed with the control diet died from natural causes between 16 and 19·5 months old. Importantly, no aged mice fed with the PEGB-enriched diet died during the same time interval, under our conditions (Fig. 1).

### Effects of age and polyphenol-rich extract from grape and blueberry-enriched diet on novel object recognition

**Fig. 2.**
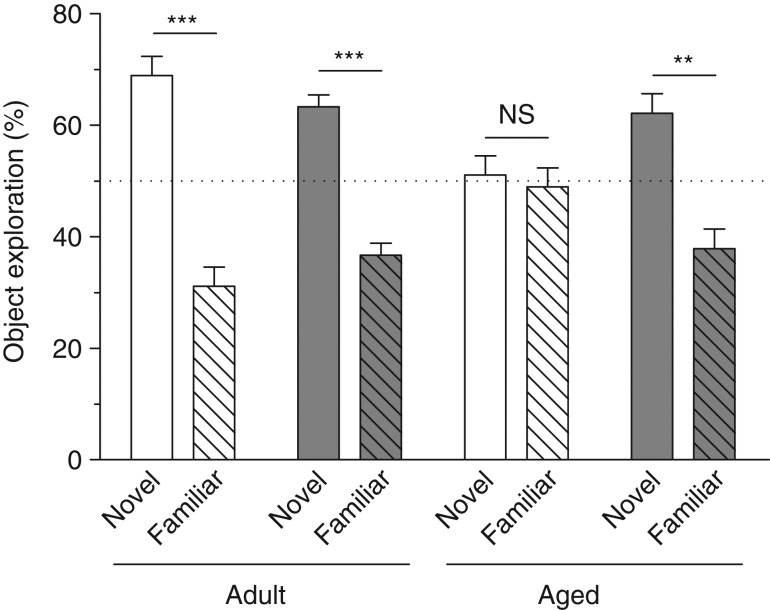
Novel object recognition memory. Percentage time spent exploring the novel object compared with percentage time spent exploring the familiar object for each group. Values are means, with their standard errors represented by vertical bars (*n* 9–10 per group). Adult mice on the control diet (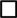) or the polyphenol-rich extract from grape and blueberry (PEGB)-enriched diet (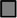) spent preferentially more time exploring the novel object than the familiar object. Aged mice on the control diet did not present a preference for the novel object. Aged mice fed the PEGB-enriched diet spent more time exploring the novel object than the familiar one, similarly to the control adult mice. ** *P* < 0·01, *** *P* < 0·001.

A mouse with a good object recognition memory was expected to spend more time exploring the novel object than the familiar one during the testing session. Thus, adult control, adult supplemented and aged supplemented, but not aged control, mice spent preferentially more time exploring the new object during the testing session (three-way ANOVA: interaction object × age × diet: *F*_(1,35)_ = 6·827, *P* = 0·0131; paired *t* test: adult control: *t* = 5·453, *P* = 0·0004; adult supplemented: *t* = 6·241, *P* = 0·0002; aged supplemented: *t* = 3·459, *P* = 0·0072; aged control: *t* = 3·459, *P* = 0·7618) (Fig. 2).

### Effects of age and polyphenol-rich extract from grape and blueberry-enriched diet on spatial learning and memory

Irrespective of diet, aged mice swam slower than adult mice, as revealed by an ANOVA on swim speed over the trials in the cued task (age effect: *F*_(1,68)_ = 5·569, *P* = 0·0212; data not shown). As the latency to reach the platform was dependent on swimming speed, the distance covered to reach the platform was used as a more appropriate criterion for evaluating the acquisition rate for the cued task and spatial learning.

All groups had similar visual capabilities and did not show any impairment in the cued version of the MWM. Indeed, all groups travelled similar distances to reach the visible platform, as revealed by the two-way ANOVA, which indicated no effect of age or diet (data not shown).

**Fig. 3.**
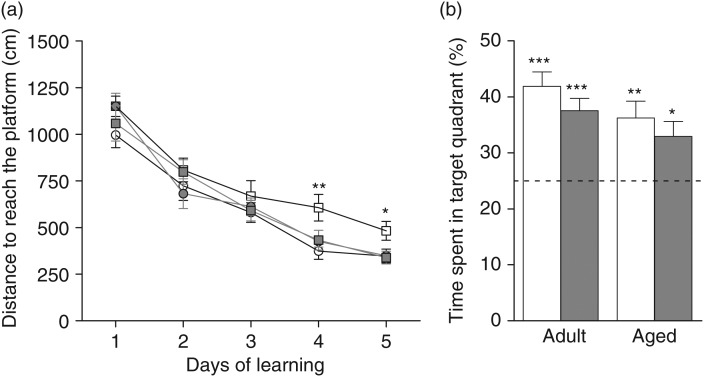
Spatial learning and memory. (a) Distance to reach the platform in adult control mice (–○–), adult mice fed the polyphenol-rich extract from grape and blueberry (PEGB)-enriched diet (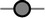), aged control mice (–□–) and aged PEGB-enriched diet-fed mice (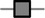). Values are means, with their standard errors represented by vertical bars (*n* 14–20 per group). Performance differed significantly on days 4 and 5 of the learning phase (* *P* < 0·05, ** *P* < 0·01 *v.* adult control). (b) Percentage of time spent by control mice (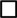) and PEGB-enriched diet-fed mice (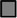) in the target quadrant during the probe test. The dotted line corresponds to chance level (25 %). Values are means, with their standard errors represented by vertical bars (*n* 14–20 per group). All groups remembered the platform location, as they spent more time in the target quadrant (* *P* < 0·05, ** *P* < 0·01, *** *P* < 0·001 *v.* chance level).

After 1 d, mice were trained in the spatial version of the MWM to test the effects of polyphenols on spatial learning and memory. As shown in Fig. 3(a), the distance to reach the platform decreased significantly over the 5 d, indicating that all groups learned the platform location (day effect: *F*_(4,272)_ = 91·51; *P* < 0·0001). Moreover, a three-way ANOVA, followed by a *post hoc* analysis, revealed that aged control mice travelled significantly longer distances to find the platform than adult control and supplemented mice, thus revealing an age-related spatial learning deficit (interaction age × diet: *F*_(1,68)_ = 4·662, *P* = 0·0345; adult control *v.* aged control: *P* = 0·002; adult supplemented *v.* aged control: *P* = 0·0247). However, aged mice that received the PEGB-enriched diet performed better than aged mice on the control diet (aged supplemented *v.* aged control: *P* = 0·0382). No diet effect was observed in adult mice. A separate analysis on each day revealed that aged mice swam significantly longer distances to reach the platform than the other groups on days 4 and 5 (day 4: adult control *v.* aged control: *P* = 0·0018, adult supplemented *v.* aged control: *P* = 0·0137, aged supplemented *v.* aged control: *P* = 0·0291; day 5: adult control *v.* aged control: *P* = 0·0125, adult supplemented *v.* aged control: *P* = 0·0137, aged supplemented *v.* aged control: *P* = 0·0144) (Fig. 3(a)).

At 48 h after the last training session, spatial memory was evaluated by a probe test. A one-sample test compared with the chance level (25 %) revealed that all groups spent significantly more time in the target quadrant (adult control: *t* = 6·587, *P* < 0·0001; adult supplemented: *t* = 5·766, *P* < 0·0001; aged control: *t* = 3·768, *P* = 0·0013; aged supplemented: *t* = 2·942, *P* = 0·0114). Furthermore, a two-way ANOVA performed on the percentage of time spent in the target quadrant revealed no age or diet effects and no age × diet interaction (*F*_(1,68)_ = 0035), suggesting that all four groups remembered the location of the platform equally well (Fig. 3(b)). The two-way ANOVA performed on the number of target annulus crossings only revealed an age effect and no diet effect nor age × diet interaction (data not shown).

### Effects of age and polyphenol-rich extract from grape and blueberry-enriched diet on hippocampal gene expression

**Fig. 4.**
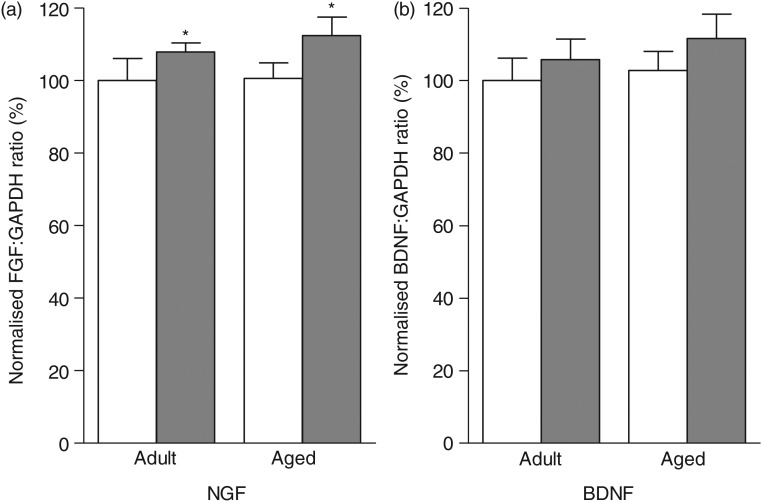
mRNA expression of hippocampal plasticity-related genes in control mice (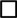) and polyphenol-rich extract from grape and blueberry (PEGB)-enriched diet-fed mice (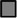). Values are means, with their standard errors represented by vertical bars (*n* 10 per group). (a) PEGB supplementation significantly increased hippocampal nerve growth factor (NGF) mRNA expression in both adult and aged mice (* *P* < 0·05, diet effect). (b) Hippocampal brain-derived neurotrophic factor (BDNF) mRNA expression was not modified by age or polyphenol supplementation. GAPDH, glyceraldehyde-3-phosphate dehydrogenase.

To study the neurobiological mechanisms underlying the behavioural improvements in aged polyphenol-fed mice, we examined whether age and a polyphenol-enriched diet modulated the hippocampal expression of some plasticity genes, particularly focusing on neurotrophins, such as *Bdnf* and *Ngf*. A two-way ANOVA performed on the hippocampal mRNA expression of *Ngf* revealed a diet effect (*F*_(1,36)_ = 4·402; *P* = 0·043); indeed, an increase in mRNA *Ngf* levels was observed in both adult and aged mice fed on the PEGB-enriched diet (Fig. 4(a)). However, no effect of age or diet on the *Bdnf* mRNA levels was detected (diet: *F*_(1,36)_ = 1·491, *P* = 0·23; age: *F*_(1,36)_ = 0·526, *P* = 0·4729) (Fig. 4(b)).

### Effects of age and polyphenol-rich extract from grape and blueberry-enriched diet on hippocampal neurogenesis

**Fig. 5.**
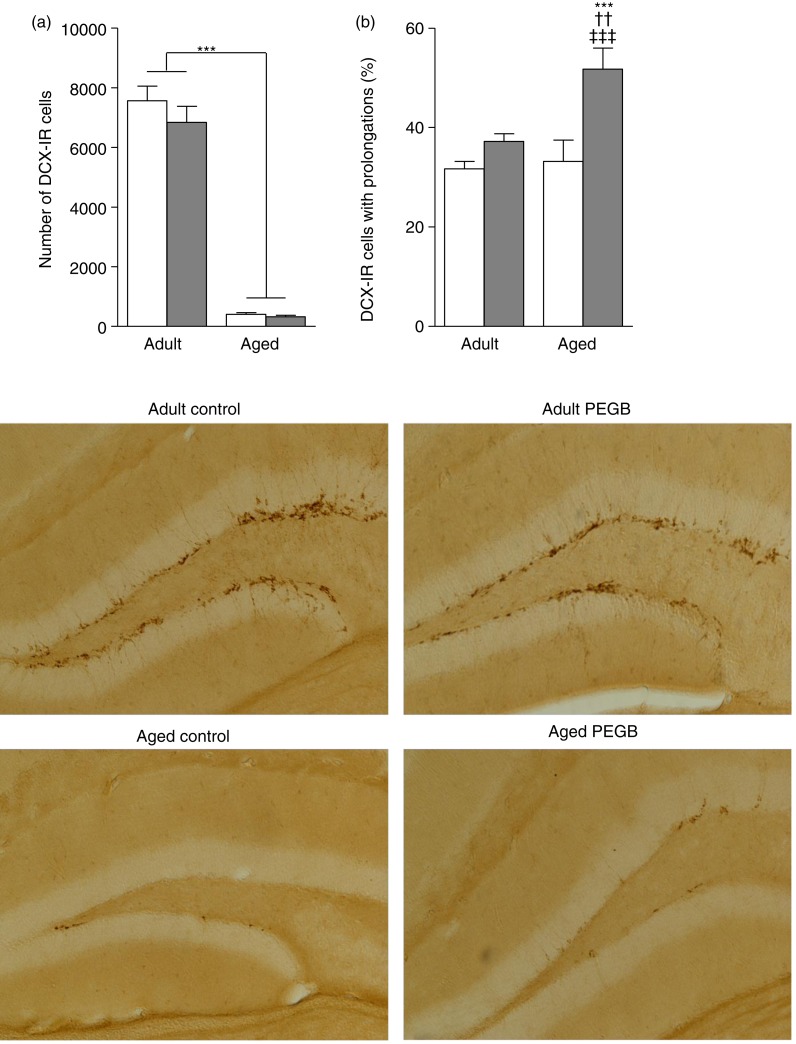
Hippocampal adult neurogenesis in control mice (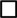) and polyphenol-rich extract from grape and blueberry (PEGB)-enriched diet-fed mice (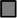). Values are means, with their standard errors represented by vertical bars (*n* 9–10 per group). (a) Analysis of the number of doublecortin-immunoreactive (DCX-IR) cells in the hippocampus. The number of newly generated immature neurons in the hippocampus decreased significantly with age (*** *P* < 0·0001, age effect). (b) Aged mice on the PEGB-enriched diet had a greater proportion of newly generated immature neurons with prolongations than the other groups (*** *P* < 0·0001 *v.* adult control; †† *P* < 0·01 *v.* adult PEGB; ‡‡‡ *P* < 0·001 *v.* aged control).

A two-way ANOVA on the number of DCX-IR cells revealed a highly significant age effect (*F*_(1,34)_ = 340·491; *P* < 0·0001), but no diet effect (*F*_(1,34)_ = 1·167; *P* = 0·2876) or age × diet interaction (*F*_(1,34)_ = 0·760; *P* = 0·3896), indicating that, irrespective of diet, aged mice have fewer newly generated immature neurons than adult mice (Fig. 5(a)). However, a two-way ANOVA analysis on the number of DCX-IR cells with dendritic prolongations, indicative of cell maturation level, revealed age (*F*_(1,34)_ = 6·375; *P* = 0·0164) and diet (*F*_(1,34)_ = 14·278; *P* = 0·0006) effects, and an age × diet interaction (*F*_(1,34)_ = 4·179; *P* = 0·0487). Fisher's *post hoc* analysis showed that aged mice fed the PEGB-enriched diet had a greater proportion of newly generated immature neurons with prolongations than the other groups (adult control *v.* aged supplemented: *P* = 0·0001; adult supplemented *v.* aged supplemented: *P* = 0·0027; aged control *v.* aged supplemented: *P* = 0·0002) (Fig. 5(b)).

## Discussion

The aim of the present study was to assess the effectiveness of a polyphenol-rich extract from grape and blueberry in preventing age-related cognitive decline in mice and to investigate potential neurobiological mechanisms underlying the behavioural observations.

There is a certain level of controversy over whether polyphenols and/or their active metabolic derivatives^(^^18^^)^ reach the central nervous system, to exert their effects on brain functions. After only 6 weeks of a PEGB-enriched diet, some flavan-3-ols and their conjugated metabolites were detected in mouse brain, suggesting that polyphenol neuroprotective effects may be due to direct neurobiological action. According to previous studies studying the bioavailability of the same grape and blueberry combination (PEGB) in mice^(^^9^^,^^14^^)^, these metabolites are also present in plasma (data not shown). Still, our results are in agreement with previous reports of detectable levels of flavan-3-ols, anthocyanins and resveratrol in brain tissue after oral ingestion^(^^18^^)^. The existence of specific polyphenol binding sites and receptors at the cell plasma membrane level in rat brain has even been suggested to explain a direct neurobiological brain action^(^^19^^)^.

Interestingly, we observed that mice fed the PEGB-enriched diet had a higher survival rate than controls; this result is consistent with other reports that some polyphenols like resveratrol increased life expectancy by modulating sirtuin expression^(^^20^^)^. However, in our conditions, sirtuin 1 mRNA hippocampal expression was not modified (data not shown). An overall decrease in oxidative stress by polyphenols could also explain their beneficial effect on the survival rate^(^^21^^,^^22^^)^. This observation suggested that phenolic compounds had a general ‘health effect’ that was indirectly beneficial to memory. This is coherent with studies showing that polyphenols may have beneficial effects on metabolic parameters, such as insulin sensitivity or blood pressure, for example^(^^23^^)^. Combined with results on their cerebral availability, their effect on lifespan suggests that the beneficial effects of PEGB on memory may result from both direct, i.e. central, and indirect, i.e. peripheral, actions.

Previous studies reported that polyphenols slowed or delayed age-related physiological and functional deficits^(^^4^^,^^18^^)^. These results revealed that PEGB prevented or delayed the development of age-related learning and memory deficits, since, contrary to the control aged mice, those fed with PEGB had learning and memory performances comparable with those of adults.

Data obtained in these experiments showed that PEGB consumption prevented recognition memory impairment during ageing. This was in agreement with previous results showing the beneficial effects of polyphenols on NOR in rodents, including a study by our consortium, in 3xTg-AD mice, using the same extract^(^^14^^,^^24^^,^^25^^)^. The NOR task is useful for assessing long-term object recognition memory (up to 24 h). Although the anatomical basis of this memory is still unclear, and might also involve the perirhinal cortex, the important role of the hippocampus in both recollection and familiarity processes has been well described^(^^26^^–^^28^^)^.

In order to specifically target a hippocampal effect of PEGB, we used the MWM in a task for assessing hippocampal-dependent learning and memory^(^^29^^)^. Our results confirmed that this task reveals age-related hippocampal learning and memory deficits^(^^30^^)^. Aged mice displayed spatial learning deficits as they travelled a significantly longer average distance than adults to reach the platform, especially in the last 2 d of the learning phase. This suggested that aged mice retained the ability to learn the location of the hidden platform, but were significantly slowed by ageing. Moreover, a 14-week supplementation with PEGB prevented this spatial learning deficit.

Indeed, we observed that aged mice fed the PEGB-enriched diet performed as well as control adult mice, without any observable alteration in their learning capacity. However, contrary to expectations, aged mice did not exhibit any significant spatial memory deficit, evaluated 48 h after the last day of learning. This result suggested that the retention time was too short to detect an age-related spatial memory deficit in 19-month-old mice under our conditions.

PEGB consumption induced a significant increase in hippocampal *Ngf* expression, in both adult and aged animals. Our previous results also revealed this increase in middle-aged mice^(^^8^^)^. An increase in NGF expression in mice brain in response to polyphenol consumption has already been described, using other sources and types of polyphenols (from olive pulp *Olea europaea* L.)^(^^31^^)^. Neurotrophic factors have been shown to be involved in modulating cerebral plasticity, since they are implicated in neuronal survival, outgrowth and differentiation^(^^32^^)^. NGF also plays a role in modulating synaptic plasticity and the long-term potentiation process in the hippocampus^(^^33^^)^. Moreover, increased NGF expression has been associated with an improvement in memory performance, particularly in aged animals^(^^34^^,^^35^^)^. No modification in hippocampal *Bdnf* mRNA expression due to the influence of age or polyphenols was observed under our conditions. This result is coherent with previous data obtained with the same PEGB in middle-aged mice^(^^8^^)^. However, an increased protein expression was observed in 3xTg-AD mice fed with a PEGB-enriched diet^(^^14^^)^. Up- or down-regulation of BDNF during ageing seems to be condition dependent. Thus, an increase in BDNF expression was described in the murine hippocampus during normal ageing, but not when mice displayed age-related pathological changes^(^^36^^)^. Moreover, physical activity can stimulate hippocampal BDNF expression^(^^37^^)^ and it may be supposed that, in our study, the physical activity involved in the behavioural tasks (MWM) may offset the ageing effect, thus masking any changes in BDNF expression already described in aged animals with or without polyphenol consumption^(^^38^^,^^39^^)^.

To investigate the impact of PEGB at a neurobiological level, in view of the relationship between hippocampal neurogenesis and memory and learning performance^(^^40^^)^, we evaluated adult neurogenesis in the DG of treated mice. As previously described, an age-related decrease in the number of DCX-IR cells was observed in the DG, indicating that increasing age has a strong impact on the number of new neurons^(^^41^^)^. The PEGB-enriched diet significantly modified the morphology of newborn neurons in aged mice, inducing an increase in the proportion of DCX-IR cells with prolongations. This observation may reflect enhanced survival of newly generated, immature neurons and/or better growth and differentiation. The increased proportion of newly generated neurons exhibiting dendrite prolongations induced by PEGB may be linked to the positive effect on behaviour and hippocampal-dependent learning. Hippocampal neurogenesis is directly linked to cognition^(^^42^^)^. Interestingly, a comparable observation was reported in aged rats – an increase in dendritic arborisation of DCX-IR cells, associated with improved spatial learning and memory – after a nutritional supplementation with vitamin A^(^^16^^)^. Moreover, using BrdU, Harada *et al.*^(^^43^^)^ observed an enhancement of neuron survival, in the DG of mice following resveratrol supplementation. More recently, Shukitt-Hale *et al*.^(^^44^^)^ reported a correlation between hippocampal neurogenesis (number of neurons proliferating) and working memory in rats consuming a berry diet. The positive effect of polyphenols on hippocampal neurogenesis may be linked to the up-regulation of hippocampal *Ngf* expression^(^^32^^,^^34^^,^^35^^)^, which may, thus, contribute to maintaining hippocampal-dependent learning during ageing.

### Conclusion

Few studies have investigated the beneficial effects of a combination of polyphenol-rich extracts on learning and memory. Our data show that the consumption of concentrated polyphenols, extracted from grapes and blueberries, improve brain plasticity and memory performance and may be effective to maintain cognitive functions during ageing. Although the exact mechanism is difficult to pinpoint, our data provide evidence of both direct and indirect effects on the brain. The well-being of aged people is closely linked to good memory performance and proper brain functioning, which very often deteriorate during ageing. Together, the data obtained in this study are in line with the hypothesis that optimised preventive nutrition may promote the maintenance of a satisfactory cognitive state in elderly subjects and may thus prevent or delay dementia, and contribute to a healthy ageing.

## Supplementary material

For supplementary material accompanying this paper visit https://doi.org/10.1017/jns.2018.10 click here to view supplementary material
