# Supplementary material for: Polyphenol-rich extract from grape and blueberry attenuates cognitive decline and improves neuronal function in aged mice
Source: J Nutr Sci. 2018 May 21;7:e19. doi: 10.1017/jns.2018.10 (PMC5971226; doi:10.1017/jns.2018.10)
Supplement: Supplementary file 1 [file S2048679018000101sup001.docx]

### Supplementary material

### Supplementary Table S1. Composition of control and PEGB-enriched diets

|  | Percent (%) | |
| --- | --- | --- |
| Components | Control diet | PEGB-enriched diet |
| Hydrochloric casein | 18 | 18 |
| Corn starch | 40 | 45 |
| Sucrose | 29.9 | 24.39 |
| Cellulose | 2 | 2 |
| Peanut oil | 2.5 | 2.5 |
| Rapeseed oil | 2.5 | 2.5 |
| Mineral compound 102 | 4 | 4 |
| Vitamin compound 102 (without vitamin A) | 1 | 1 |
| + DL methionine | 0.1 | 0.1 |
| + Vitamin A 5 IU/g | 5 IU/g | 5 IU/g |
| PEGB | 0 | 0.51 |
| PEGB: polyphenol-rich extract from grape and blueberry. PEGB was introduced at 0.51% in the diet pellets. | | |

### Supplementary Table S2. Primers used for LightCycler RT-qPCR

| Gene name | Nucleotide sequence 5’-3’ | Amplicon size (pb) |
| --- | --- | --- |
|  |  |  |
| *Gapdh* | F: GAACATCATCCCTGCATCCA  R: CCAGTGAGCTTCCCGTTCA | 78 |
| *Ngf* | F: ATCAAGGGCAAGGAGGTGACAG | 143 |
|  | R: GAGTTCCAGTGTTTGGAGTCGATG |  |
| *Bdnf* | F: AACCATAAGGACGCGGACTTG  R: TTGACTGCTGAGCATCACCC | 51 |
| Sequences are shown for forward (F) and reverse (R) primers. *Gapdh*, glyceraldehyde-3-phosphate dehydrogenase-like; *Ngf*, Nerve growth factor; *Bdnf*, brain derived neurotrophic factor. | | |
